# Anti-bacterial activity of *Corchorus olitorius* L. and *Acmella caulirhiza* Del. on *Streptococcus mutans*, a cariogenic bacterium

**DOI:** 10.4314/ahs.v21i4.23

**Published:** 2021-12

**Authors:** Hadijja Namwase, Florence Najjuka, Godfrey Bbosa

**Affiliations:** 1 Department of Pharmacology, Makerere University, P.O Box 7072, Kampala, Uganda; 2 Department of Microbiology, Makerere University, P.O Box 7072, Kampala, Uganda; 3 Department of Pharmacology, Makerere University, P.O Box 7072, Kampala, Uganda

**Keywords:** Antibacterial activity, Minimum inhibitory concentration, Plant extracts, *Corchorus olitorius* L., *Acmella caulirhiza* Del., *Streptococcus mutans*, Dental caries

## Abstract

**Background:**

Dental caries remains a global oral health challenge with the prevalence reported as high as 66.7% in adults. Despite the use of modern medicines, the prevalence of dental caries remains high. This has led to extensive screening of natural products particularly from plants such as *Corchorus olitorius* L. and *Acmella caulirhiza* Del. for anti-cariogenic activity.

**Aim:**

To assess the anti-bacterial activity of *Corchorus olitorius* L. and *Acmella caulirhiza* Del. on *Streptococcus mutans*, a cariogenic bacteria.

**Methods:**

Plant materials of *C. olitorius L.* and *A. caulirhiza* Del. were extracted using diethyl ether, methanol, distilled water by cold maceration. Agar well diffusion method was used for sensitivity and susceptibility tests on *S. mutans* (ATCC 6519).

**Results:**

The aqueous plant extract of *C. olitorius* L. and the ether plant extract of *A. caulirhiza* Del. had the highest zones of inhibition (16.10mm and 12.03mm respectively) at a concentration of 1000mg/ml. The lowest MIC and MBC were 62.5mg/ml and 250mg/ml respectively.

**Conclusion:**

Both *C. olitorius* L. and *A. caulirhiza* Del. as used in oral health practices have been found to have antibacterial activity against the cariogenic *S. mutans*. Further studies should be conducted to isolate bioactive compounds against *S. mutans*.

## Introduction

Diseases of the oral cavity are a common problem globally and they mainly include dental caries and periodontitis that result from the disruption of the normal flora of the cavity 1. Dental caries is formed as a result of fermentation of carbohydrates by specific bacterial species such as *Streptococcus mutans* which produces lactic acid that dissolves the enamel. Considerable epidemiologic evidence links *Streptococcus mutans* to the pathogenesis of these dental caries 2.

Dental caries is a serious public health problem due to poor oral hygiene, inadequacy of resources such as dental health care personnel and services that have made people to seek for an alternative form of care in form of traditional medicine 3, 4. The finding of secondary metabolites such as alkaloids, flavanoids, tannins, antharaquinone that have antimicrobial properties has become one of the remarkable alternatives for treatment of dental caries 5. As a result, there is need to identity, isolate and purify these different bioactive compounds to make new drug molecules.

Different medicinal plants are being used in the treatment of dental caries for example *Lantana trifolia* (*Verbenaceae*), *Draceana fragrans* (*Agavaceae*) 6 including; *Corchorus olitorius* L and *Acmella caulirhiza* Del. However, there is insufficient scientific evidence and documentation on their antibacterial activity against *S. mutans*.

*Corchorus olitorius* L. (Tiliaceae) is a green leafy vegetable generally known as “Jute” (English) 7 “Muteere” (lusoga) and “Otigo” (Acholi). Besides the plant being used traditionally to treat dental caries, it is also used as folk remedy for pains, dysentery and enteritis in some communities. On the other hand, *Acmella caulirhiza* Del. (*Asteraceae*) is a flowering creeping plant commonly found near springs used traditionally to treat mouth ulcers, sore throat, toothache and earache. In Uganda, it is locally called Mukasa omusajja (Luganda).

*C. olitorius* L. and *A. caulirhiza* Del. have been found to have antibacterial activity against *Escherichia coli, Staphylococcus aureus* and *Bacillus pumilus* 7, 8. Therefore, there is need to assess the antibacterial activity of these plants against *S. mutans* since no study has been done despite use of the plants by the local communities in Uganda in the management of dental caries.

## Materials and methods

It was an experimental laboratory based study conducted at the Department of Pharmacology and Therapeutics, College of Health Sciences and at the Department of Microbiology, College of Veterinary Medicine, Animal Resources and Biosecurity (COVAB), Makerere University. The leaves of *C. olitorius* L. and the aerial (leaves, stems and flowers) parts of *A. caulirhiza* Del. were used. The plants studied were selected basing on local communities' claims that they were effective in treating dental caries and also through ethnobotanical surveys. The plants were picked from Komamboga, Wakiso district during the rainy season, rinsed, dried under a solar drier for 14 days and pounded to produce a powder which was stored in airtight containers in a cool dry.

### Extraction of *C. olitorius* L. and *A. caulirhiza* Del

About 500 grams of *C. olitorius* L. and 400g of *A. caulirhiza* Del. were extracted using serial exhaustive extraction with different solvents of increasing polarity starting with diethyl ether and then methanol. The plant powders were extracted using cold maceration method^9^. The plant powders were soaked in 2.5 liters of 96% diethyl ether for 72 hours with intermittent shaking, filtered using a Whatman No. 4 filter paper and concentrated using a rotary evaporator (Rotavapor® R-210/R) to recover the diethyl ether. The remaining concentrated filtrate was dried to form the diethyl ether plant extract for either plants. The remaining marc was re-extracted using the recovered diethyl ether for 48 hours. The remaining marc of *C. olitorius* L. and *A. caulirhiza* Del. was dried and later soaked in 2.5 liters of 96% methanol for 72 hours with intermittent shaking, filtered and concentrated using a rotary evaporator to recover the methanol and form the methanol plant extract for either plants. The remaining marc was re-extracted using the recovered methanol for 48 hours. The diethyl ether and methanolic filtrates were mixed in a proportion of 1:1 and were later concentrated using a rotary evaporator to form *C. olitorius* L. and *A. caulirhiza* Del. total crude plant extracts respectively.

About 100 grams of new dried plant material of *C. olitorius* L. and *A. caulirhiza* Del. were mixed and soaked in 1 liter of boiled distilled water for 6 hours, filtered and concentrated using a freeze drier to form an aqueous plant extract of *C. olitorius* L. and *A. caulirhiza* Del. respectively. The different dried solvent extracts were stored under refrigeration at 4°C.

Finally, about 1 gram of each of the diethyl ether, methanol, aqueous and total crude plant extracts of *C. olitorius* L. and *A. caulirhiza* Del. were weighed and dissolved in a few drops of dimethylsulfoxide (DMSO) until dissolution and then topped up with sterilized distilled water to make 1ml for the diethyl ether, methanolic and total crude extracts to make a stock solution of 1000mg/ml. 1ml of sterilized distilled water was used for the aqueous extracts to make a stock solution of 1000mg/ml.

### Microbial strain of *Streptococcus mutans*

The American Type Culture Collection strain (ATTC 6519) of *S. mutans* was used. It was sub cultured on 5% sheep blood agar (pH 7.3). The plates were placed in an anaerobic jar (Oxoid, Britain). An anaerobic gas pack (Biomerieux manufacturer, France) and an indicator (Oxoid manufacturer, Britain) were also placed in the anaerobic jar. These were then incubated at 37°C for 24hrs. Using direct colony suspension method, a 0.5 Mcfarland standard of *S. mutans* (ATCC 6519) was prepared and its turbidity compared with the 0.5 Mcfarland standard 10. The adjusted inocula equivalent to 1.5 x 10^8^ cfu/mL was used in the different bioassay procedures.

### Screening for antibacterial activity

Antimicrobial activity of the solvent extracts was determined by using the agar well diffusion method. Sterile plates of solidified mueller hinton agar were inoculated with two loopfuls of freshly prepared inocula equivalent to 1.5 x 10^8^ cfu/mL to form a cross and streaked to form a mat of the test microorganism. Each plate was divided into four quadrats and each quadrat was labelled. Four wells (2-solvent leaf extracts, a positive and negative control) of 6 mm diameter were bored in the agar using a sterilized cork borer. About 50µL of 1000mg/ml of the different solvent plant extracts were introduced into each well using a micropipette. Ciprofloxacin was used as the positive control as it a broad spectrum antibiotic active against gram positive and gram negative bacteria, DMSO was used as the negative control for diethyl ether, methanol, total crude extracts and sterilized distilled water was used as the negative control for aqueous extracts. The plates were inserted in an upright position in an anaerobic jar (Oxoid manufacturer, Britain) and incubated anaerobically at 37 ºC for 48hrs as described by Jain et al., 11 with modification. Antibacterial activity of the extracts was determined by measuring the zones of inhibition in millimeters using a Vernier caliper and read off against a millimeter rule and recorded immediately. The tests were done in duplicates.

### Determination of minimum inhibition concentration (MIC)

The MIC was determined depending on the least concentration of the extract that showed zone of clearance using the agar well diffusion method. Only the solvent plant extracts that showed antibacterial activity against *Streptococcus mutans* were used to determine MIC.

Using double dilution method, the stock solution of 1000mg/ml concentration was used to prepare concentrations of 500mg/ml, 250mg/ml, 125mg/ml, 62.5mg/ml, 31.25 mg/ml, 15.625mg/ml, 7.8125mg/ml, 3.906mg/ml of C. olitorius L. and A. caulirhiza Del. (12) with modification.

### Determination of the minimum bactericidal concentration (MBC)

The MBC was the minimum concentration at which no bacterial growth was observed. Only the concentrations of the different solvent plant extracts that showed zone of clearance were used to determine MBC. Petri dishes with MHA were then inoculated with 2 loopfuls of a combination of trypticasein soy broth, inoculate and extract that had previously been incubated anaerobically for 24 hours. The petri dishes were later incubated anaerobically at 37°C for 24 hours. The plate with the least concentration of the herbal extract that had no growth of bacteria was taken as the MBC for the different solvent plant extracts.

### Statistical analysis

Standard error of deviation was calculated and used to determine the 95% confidence interval using Statistical Package for Social Science version 16, Analysis of Variance (ANOVA) and Tukey's (Honest significant difference) test were used.

## Results

### Antibacterial activity of the plant extracts of *Corchorus olitorius* L. and *Acmella caulirhiza* Del. against S. mutans

Using the agar well diffusion method, all the solvent extracts of *C. olitorius* L. and *A. caulirhiza* Del. showed antibacterial activity against S. mutans. The aqueous plant extract of *C. olitorius* L. and the diethyl ether plant extract of *A. caulirhiza* Del. had the highest antibacterial activity as shown by their zones of inhibition at a concentration of 1000mg/ml. Using 6mm diameter of inhibition as the cut off, there was significant inhibition from most of the solvent extracts except the methanolic and total crude extracts of *A. caulirhiza* Del. Ciprofloxacin which was used as the positive control also showed sensitivity. DMSO and sterilized distilled water which were used as negative controls did not have any antibacterial activity (Table 1).

**Table 1:** Antibacterial activity of *Corchorus olitorius* L. and *Acmella caulirhiza* Del. against *Streptococcus mutans* at 1000mg/ml concentration for each solvent

Medicinal plant	Zone of inhibition at 1000mg/ml concentration for each solvent extract (mm)						ANOVA
		
	Aqueous	Methanol	Diethyl ether	Total crude	Ciprofloxacin	DMSO D/water	p value
***C. olitorius* L.**	22.10±0.17^a^,^b^	15.03±0.05^a^,^b^	14.03±0.05^a^,^b^*	14.03±0.05^a^,^b^*	49.13±0.10^a^	0±0.00^b^	2.25e^-16^
***A.*** ***caulirhiza* Del.**	15.03±0.05^a^,^b^	10.03±0.05^a^,^b^*	18.03±0.05^a^,^b^	10.03±0.05^a^,^b^*	49.13±0.10^a^	0±0.00^b^	2.22e^-16^

Using Tukey's HSD test at p<0.05 to detect difference between two groups

a difference detected compared to ciprofloxacin

b difference detected compared to either DMSO or distilled water

* difference not statistically significant between the two

### Minimum inhibitory concentration of *Corchorus olitorius* L. and *Acmella caulirhiza* Del. solvent extracts against *Streptococcus mutans*

Using agar well diffusion method, the minimum concentration of each solvent plant extract that showed the least zone of inhibition was determined as the MIC. The diethyl ether extracts of both *C. olitorius* L. and *A. caulirhiza* Del. had the lowest MIC of 62.5mg/ml (Table 2).

**Table 2:** Minimum inhibitory concentration (MIC) of *Corchorus olitorius* L. and *Acmella caulirhiza* Del. solvent extracts against *Streptococcus mutans*

Medicinal plant	Minimum inhibitory concentration (mg/ml) for each solvent extract						ANOVA
	
	Aqueous	Methanol	Diethyl ether	Total crude	Ciprofloxa-cin	DMSO D/water	p-value value
***C. olitorius* L.**	125.0±0.057^a^,^b^*	500.0±0.057^a^,^b^	62.5±0.057^a^,^b^	125.0±0.057 a,b*	0.004±0.05^a^	0±0.00^b^	0.00
***A.*** ***caulirhiza* Del.**	125.0±0.005^a^,^b^	500.0±0.057^a^,^b^*	62.5±0.057^a^,^b^	500.0±0.057^a^,^b^*	0.004±0.05^a^	0±0.00^b^	2.22e^-17^

Using Tukey's HSD test at p<0.05 to detect difference between two groups

a difference detected compared to ciprofloxacin

b difference detected compared to either DMSO or distilled water

* difference not statistically significant between the two.

### Minimum bactericidal concentration of *Corchorus olitorius* L. and *Acmella caulirhiza* Del. solvent extracts against *Streptococcus mutans*

Only the concentrations that showed zones of inhibition were used to determine MBC. All solvent extracts of *C. olitorius* L. had a similar MBC of 250mg/ml and the aqueous extract of A. caulirhiza Del. had the lowest MBC of 250mg/ml (Table 3).

**Table 3:** Minimum bactericidal concentration (MBC) of *Corchorus olitorius* L. and *Acmella caulirhiza* Del. solvent extracts against *Streptococcus mutans*

Medicinal plants	Minimum bactericidal concentration (mg/ml) for each solvent extract						ANOVA
	
	Aqueous	Methanol	Diethyl ether	Total crude	Ciprofloxacin	DMSO D/water	p value
***C. olitorius* L.**	250.0±0.00^a^,^b^*	250.0±0.00^a^,^b^*	250.0±0.00^a^,^b^*	250.0±0.00^a^,^b^*	0.005±0.05 a	0±0.00^b^	1.11e^-16^
***A.*** ***caulirhiza* Del.**	250.0±0.00^a^,^b^	500.0±0.05^a^,^b^*	500.0±0.05^a^,^b^*	500.0±0.05^a^,^b^*	0.005±0.05^a^	0±0.00^b^	1.0e^-7^

Using Tukey's HSD test at p<0.05 to detect difference between two groups

a difference detected compared to ciprofloxacin

b difference detected compared to either DMSO or distilled water

* difference not statistically significant between the two.

## Discussion

In this study, it was observed that the aqueous, methanolic, diethyl ether and total crude extracts of both *C. olitorius* L. and *A. caulirhiza* Del. had antibacterial activity against *S. mutans* at a concentration of 1000mg/ml (Table 1). The difference in the antibacterial activity in terms of zone of inhibition of all solvent extracts of both *C. olitorius* L. and *A. caulirhiza* Del., was significantly greater than that of DMSO and distilled water. However, the antibacterial activity of the solvent extract of *C. olitorius* L. and *A. caulirhiza* Del., was less than that of ciprofloxacin. This could be due to the difference in purity since the plant extracts were used in their crude form. There was a difference in the MIC of the aqueous, diethyl ether, ciprofloxacin, DMSO and distilled water. In the determination of MBC, the difference in the MBC of all solvent plant extracts of *C. olitorius* L. was not significant. The aqueous extract of *A. caulirhiza* Del. had an MBC which was lower than the other plant solvent extracts. This difference was statistically significant.

Using the 6mm diameter of inhibition as the cut off, the aqueous extract of *C. olitorius* L demonstrated the highest antibacterial activity with a zone of inhibition of 22.10mm (Table 1). This result correlates with reports in a differenttudy 13, in which it was demonstrated that *C. olitorius* L. had good antimicrobial activity against *E. coli, S. aureus, Y. enterocolitica, Geotrichum candidum* and *Botrytis cinerea*. The antibacterial activity of *C. olitorius* L. is due to the presence of phytochemical compounds^14^ including triterpenes, alkaloids, coumarins, flavonoids and anthraquinone that possess antibacterial activity 15,16. This therefore, supports the antibacterial activity of *C. olitorius* L. against *S. mutans* in this study.

The methanolic and crude extracts of *A. caulirhiza* Del. had the lowest antibacterial activity (Table 1). *A. caulirhiza* Del. has also been reported to possess antibacterial activity against *Escherichia coli, Staphylococcus aureus* and *Bacillus pimulus* 17. *A. caulirhiza* Del. is also reported to contain flavonoids, triterpenoids, alkylamides, alkaloids, amino acids, tannins and phenolic compounds 18. These phytochemicals present in the Acmella family influence its antibacterial activity 19. *A. caulirhiza* Del. is also reported to contain spilanthol, Undeca-2E,7Z,9E-trienoic acid isobutylamide, Undeca-2E-en-8,10-diyonic acid isobutylamide, *β*-Sitosterol, Stigmasterol, *α* and *β*-Amyrin. The phytochemical compounds present in *A. caulirhiza* Del. could be responsible for its antibacterial activity against *S. mutans*.

Of the plant extracts that had the highest zone of inhibition, the aqueous extract of *C. olitorius* L. was more active as compared to the diethyl ether extract of *A. caulirhiza* Del. (Table 1).

The diethyl ether extracts of both plants had the highest ability to inhibit growth of *S. mutans* possibly because the diethyl ether could have extracted phytochemical compounds with higher bacteriostatic properties.

*C. olitorius* L. had a higher ability to kill *S. mutans* as compared to *A. caulirhiza* Del. as portrayed by its MBC of 250mg/ml hence having higher bactericidal properties (Table 3). Only the aqueous extract of *A. caulirhiza* Del. was comparable to *C. olitorius* L.'s bactericidal activity.

In addition, the aqueous extract in both plants had similar MIC and MBC values hence had similar bacteriostatic and bactericidal activities respectively. Therefore, the aqueous extract had the best ability to efficiently extract antibacterial phytochemicals with bactericidal activity against *S. mutans*. The findings in this study are in line with those of another study^20^, where the aqueous extract of *Dissotis thollonii Cogn*. demonstrated better antibacterial activity against *S. aureus, Shigella flexneri, E. coli, Salmonella typhi* and *Enterobacter aerogenes* as compared to the methanolic extract. This therefore, supports the traditional use of decoctions of these plants in dental caries.

The therapeutic effects of plant materials generally result from a combination of secondary metabolites. Different phytochemical compounds found in *C. olitorius* L. and *A. caulirhiza* Del. exhibit different antibacterial mechanisms. For instance, flavonoids inhibit nucleic acid synthesis, cytoplasmic membrane function and energy metabolism. Tannins are reported to inhibit the growth of microorganisms by precipitating the microbial protein and thus depriving them of nutritional proteins needed for their growth and development 21. Alkaloids are reported to inhibit nucleic acid protein and membrane phospholipid biosynthesis 12. The Phenolic compounds found in *A. caulirhiza* Del. inhibit growth of microorganisms through enzyme inhibition by the oxidized compounds or through more nonspecific interactions with the proteins 22. Therefore, the existence of these different phytochemical compounds in *C. olitorius* L. and *A. caulirhiza* Del. contributes to their antibacterial activity against *S. mutans*, a cariogenic bacteria.

β-Sitosterol found in *A. caulirhiza* Del. has been reported to have antibacterial activity against different bacterial species including *S. aureus* and *E. coli* 23. This could partly explain the antibacterial activity of *A. caulirhiza* Del. against *S. mutans* which is also a gram positive bacteria as *S. aureus. A. caulirhiza* Del. also containsifferent alkylamides including spilanthol (N-isobutyldecatriene-2,6,8-amide) 24. These amides may directly inhibit bacterial pathogens and also provide localised pain relief to patients 24. *A. caulirhiza* Del. is used traditionally in treating toothache, stomachache and earache, this could also be attributed to the alkylamides that provide an analgesic effect 18.

Therefore, the observed antibacterial activity against *S. mutans* validates the traditional use of *C. olitorius* L. and *A. caulirhiza* Del. in treatment of decayed teeth, gingivitis, toothache or wounds in the mouth. As a result, these plants could be potential sources of new antibiotic compounds.

## Conclusion

This study has proved that *C. olitorius* L. and *A. caulirhiza* Del. possess antibacterial activity against the cariogenic bacteria *S. mutans* which is implicated in the initiation of dental caries. This study is therefore in agreement with the use of these traditional medicinal plants in the prevention and treatment of dental caries. Isolation and purification of phytochemical compounds from these plants may yield significant novel plant based antimicrobials that can have activity against other cariogenic micro-organism.

## Limitations

This study only looked at a laboratory-based specie of *S. mutans*. I suggest that further research be done on other cariogenic bacteria like *S.Sobrinus* and *Lactobacillus* which propagate thecaries when *S.mutans* initiates it. In addition, studying the local *S.mutans* from caries in the local communities not the lab species can be done.
